# Structure-Guided Computational Approaches to Unravel Druggable Proteomic Landscape of *Mycobacterium leprae*

**DOI:** 10.3389/fmolb.2021.663301

**Published:** 2021-05-07

**Authors:** Sundeep Chaitanya Vedithi, Sony Malhotra, Marta Acebrón-García-de-Eulate, Modestas Matusevicius, Pedro Henrique Monteiro Torres, Tom L. Blundell

**Affiliations:** ^1^Department of Biochemistry, University of Cambridge, Cambridge, United Kingdom; ^2^Rutherford Appleton Laboratory, Science and Technology Facilities Council, Oxon, United Kingdom; ^3^Laboratório de Modelagem e Dinâmica Molecular, Instituto de Biofísica Carlos Chagas Filho, Universidade Federal do Rio de Janeiro, Rio de Janeiro, Brazil

**Keywords:** *Mycobacterium leprae*, amino acid substitution, chokepoint reactions, drug binging sites, homology (comparative) modeling, protein interface

## Abstract

Leprosy, caused by *Mycobacterium leprae (M. leprae)*, is treated with a multidrug regimen comprising Dapsone, Rifampicin, and Clofazimine. These drugs exhibit bacteriostatic, bactericidal and anti-inflammatory properties, respectively, and control the dissemination of infection in the host. However, the current treatment is not cost-effective, does not favor patient compliance due to its long duration (12 months) and does not protect against the incumbent nerve damage, which is a severe leprosy complication. The chronic infectious peripheral neuropathy associated with the disease is primarily due to the bacterial components infiltrating the Schwann cells that protect neuronal axons, thereby inducing a demyelinating phenotype. There is a need to discover novel/repurposed drugs that can act as short duration and effective alternatives to the existing treatment regimens, preventing nerve damage and consequent disability associated with the disease. *Mycobacterium leprae* is an obligate pathogen resulting in experimental intractability to cultivate the bacillus *in vitro* and limiting drug discovery efforts to repositioning screens in mouse footpad models. The dearth of knowledge related to structural proteomics of *M. leprae*, coupled with emerging antimicrobial resistance to all the three drugs in the multidrug therapy, poses a need for concerted novel drug discovery efforts. A comprehensive understanding of the proteomic landscape of *M. leprae* is indispensable to unravel druggable targets that are essential for bacterial survival and predilection of human neuronal Schwann cells. Of the 1,614 protein-coding genes in the genome of *M. leprae*, only 17 protein structures are available in the Protein Data Bank. In this review, we discussed efforts made to model the proteome of *M. leprae* using a suite of software for protein modeling that has been developed in the Blundell laboratory. Precise template selection by employing sequence-structure homology recognition software, multi-template modeling of the monomeric models and accurate quality assessment are the hallmarks of the modeling process. Tools that map interfaces and enable building of homo-oligomers are discussed in the context of interface stability. Other software is described to determine the druggable proteome by using information related to the chokepoint analysis of the metabolic pathways, gene essentiality, homology to human proteins, functional sites, druggable pockets and fragment hotspot maps.

## Introduction

*Mycobacterium leprae* causes leprosy in about 200,000 people each year globally. Leprosy is a dermato-neurological infectious disease with varied clinical manifestations, often resulting in peripheral sensorimotor/demyelinating neuropathy leading to permanent nerve damage and disability. The World Health Organization currently recommends a combinatorial therapy [multidrug therapy (MDT)] with Dapsone, Rifampicin (Rifampin) and Clofazimine to treat leprosy (Manglani and Arif, 2006). MDT has proven effective in reducing the prevalence and controlling the incidence from about 5 million new cases in the 1990s to ∼200,000 new cases from the year 2005 (after India declared the elimination of leprosy). However, with the emergence of single and multidrug-resistant strains of *M. leprae*, novel therapies are essential to curb ongoing transmission of the disease. Also, the current therapy duration with MDT is 1 year leading to reduced treatment compliance and increased defaulter rates globally (Cambau et al., 2018).

*Mycobacterium leprae* is phylogenetically the closest bacterial species to *Mycobacterium tuberculosis (M. tuberculosis).* However, the *M. leprae* has a reduced genome of 3.2 Mbp, compared to 4.4 Mbp in *M. tuberculosis*, and survive with only 1,614 protein coding genes which are largely annotated based the features of their homologues in *M. tuberculosis* and other mycobacterial species (Cole et al., 2001). Dapsone interacts with bacterial dihydropteroate synthase, an enzyme essential for folic acid biosynthesis in bacteria. It is absent in humans (Cambau et al., 2006). Rifampin interacts with RNA polymerase, an enzyme critical for DNA dependent RNA synthesis (transcription) in *M. leprae*. These drugs are either bacteriostatic or bactericidal. However, they do not interfere with predilection of *M. leprae* for human nerve cells, which is a significant cause for demyelinating neuropathy in leprosy (Lockwood and Saunderson, 2012). Newer antibacterial agents that can effectively treat the disease within a short duration of time and prevent nerve damage are essential to reduce morbidity associated with the disease (Rao and Jain, 2013). Currently known drugs for leprosy, their drug target proteins and references related to their mechanisms of action are listed in Table 1.

**TABLE 1:** Drugs and their corresponding target proteins in *M. leprae.*

**Drug**	**Target proteins/Ribosomal subunits**	**Gene (gene name)**	**References**
Dapsone	Dihydropteroate synthase (DHPS)	*folP1* (ML0224)	Williams et al., 2000
Rifampin	β-subunit of the DNA-dependent RNA polymerase	*rpoB* (ML1891)	Lin et al., 2017
Clofazimine	Unknown	-	Lechartier and Cole, 2015
Fluoroquinolones	DNA gyrase subunit A	*gyrA* (ML0006)	Blower et al., 2016
	DNA gyrase subunit B	*gyrB* (ML0005)	Yamaguchi et al., 2016
Macrolides	50S subunit (23S rRNA in particular)	-	Ji et al., 1996
Minocycline	30S ribosomal subunit, blocking the binding of aminoacyl-tRNA to the 16S rRNA	-	Ji et al., 1996
Thioamides	Enoyl-ACP-reductase	*inhA* (ML1806)	Wang et al., 2007
Bedaquiline	Proton pump of ATP synthase	*atpE* (ML1140)	Guo et al., 2021
Epiroprim	Dihydrofolate reductase	*folA* (ML1518)	Dhople, 2002

Knowledge of the structural components of the proteome of *M. leprae* is critical for identifying drug target proteins and deciphering their essential roles in the survival of the pathogen. Key enzymes that catalyze chokepoint reactions can act as potential drug targets for antimycobacterial discovery. However, information related to 3D structures of these proteins is scarce for *M. leprae*, necessitating a more focussed structural genomics effort to unravel the druggable proteomic landscape of this bacillus long known to humankind.

Software tools that predict stability and affinity changes in drug-target proteins due to substitution mutations are discussed in the context of antimicrobial resistance. While the emphasis is on deciphering the druggable proteome, we provide a brief overview of the structure-guided virtual screening tools and methods that facilitate the chemical expansion of fragment-like small molecules to lead-like or drug-like compounds in the active or allosteric sites of the target protein.

### Proteome Modeling in *Mycobacterium leprae* and Its Relevance to Structure-Guided Drug Discovery

Of the 1,614 annotated genes that are expressed in *M. leprae*, the structures of only 17 proteins are available (see Table 2) to date in the publicly available databases [Protein Data Bank (PDB) (Berman et al., 2000)], as opposed to around 1,277 entries for *Mycobacterium tuberculosis*. Solving the crystal/cryoEM structures of all the potential drug targets in *M. leprae* requires costly and labor intensive effort. Given the high sequence identity of many of the *M. leprae* proteins with their homologous counterparts in *M. tuberculosis* with solved structures in the PDB, employing computational tools to perform comparative modeling of proteins in *M. leprae* can be a robust alternative for acquiring a preliminary understanding of the functional sites and small molecule interactions.

**TABLE 2:** List of protein structures available for *M. leprae* in Protein Data Bank

**Gene Id**	**PDB Id**	**Description**	**References**
ML2441	4EO9	Crystal structure of a phosphoglycerate mutase gpm1 from *Mycobacterium leprae*	Baugh et al., 2015
ML0210	4ECP	X-ray crystal structure of Inorganic Pyrophosphate PPA from *Mycobacterium leprae*	Unpublished
ML0560	4J07	Crystal structure of a PROBABLE RIBOFLAVIN SYNTHASE, BETA CHAIN RIBH (6,7-dimethyl-8-ribityllumazine synthase, DMRL synthase, Lumazine synthase) from *Mycobacterium leprae*	Unpublished
ML1382	5IE8	The pyrazinoic acid binding domain of Ribosomal Protein S1 from Mycobacterium tuberculosis*	Huang B. et al., 2016
ML0482	1BVS	RUVA Complexed to a Holliday Junction	Roe et al., 1998
ML2684	3AFP	Crystal structure of the single-stranded DNA binding protein from *Mycobacterium leprae* (Form I)”	Kaushal et al., 2010
ML2640	2CKD	Crystal structure of ML2640 from *Mycobacterium leprae*	Graña et al., 2007
ML0380	1LEP	Three-Dimensional Structure of the Immunodominant Heat-Shock Protein Chaperonin-10 of *Mycobacterium Leprae*	Mande et al., 1996
ML1962	3I4O	Crystal Structure of Translation Initiation Factor 1 from Mycobacterium tuberculosis*	Hatzopoulos and Mueller-Dieckmann, 2010
ML2428A	5O61	The complete structure of the *Mycobacterium smegmatis* 70S ribosome*	Hentschel et al., 2017
ML1485	4WKW	Crystal Structure of a Conserved Hypothetical Protein from *Mycobacterium leprae* Determined by Iodide SAD Phasing	Unpublished
ML2174	3R2N	Crystal structure of cytidine deaminase from *Mycobacterium leprae*	Baugh et al., 2015
ML1806	2NTV	*Mycobacterium leprae* InhA bound with PTH-NAD adduct	Wang et al., 2007
ML2684	3AFQ	Crystal structure of the single-stranded DNA binding protein from *Mycobacterium leprae* (Form II)	Kaushal et al., 2010
ML2069	4EX4	The Structure of GlcB from *Mycobacterium leprae*	Unpublished
ML2640	2UYO	Crystal structure of ML2640c from *Mycobacterium leprae* in an hexagonal crystal form	Graña et al., 2007
ML2640	2UYQ	Crystal structure of ML2640c from *Mycobacterium leprae* in complex with S-adenosylmethionine	Graña et al., 2007

*The solved region of the protein structure is 100% in sequence identity with *M. leprae.*

Different groups have made several attempts to model the proteins of *M. leprae*. Table 3 lists two web-resources where such information is available. Computational protein structure modeling can reduce the sequence-structure gaps and enable genome-scale modeling of infectious pathogens (Bienert et al., 2017). Although the paucity of structural proteomics information for *M. leprae* in the publicly available databases is a challenge, the software developed in the Blundell laboratory will be useful in performing proteome scale modeling pipeline (*Vivace*) for proteomes of Mycobacterial pathogens and other bacterial species (Skwark et al., 2019). *Vivace* optimizes template selection, enables sequence-structure alignments, and constructs optimal quality models in both apo- and ligand-bound states. To facilitate multi-template modeling, protein structures from the entire PDB are initially organized in a structural profile database named TOCCATA (Ochoa-Montaño et al., 2015). Protein structures within each profile are classified based on domain annotations in CATH (Sillitoe et al., 2019) and SCOP (Andreeva et al., 2020) databases, pre-aligned and functionally annotated with information derived from UniProt (The UniProt Consortium, 2021) and PDB. The query protein sequence is aligned with representative structures from each cluster using a sequence-structure alignment tool named FUGUE (Shi et al., 2001). FUGUE recognizes distant homologues by sequence-structure comparison using environment-specific substitution tables and structure-dependent gap penalties. Alignments generated by FUGUE are fed into Modeler 9.24 (Webb and Sali, 2016) for model building. The ligands and other small molecules are modeled into corresponding protein structure models at sites recognized from the ligand-bound templates. Multiple models are generated based on the number of cluster hits, ranging from 3 to ∼1,000 models per query sequence in the *M. leprae* proteome. These models are of different states (ligand-bound and apomeric) and of varying quality based on the templates used in each profile.

**TABLE 3:** Web resources with models of *M. leprae* proteins (modelled using single templates).

**Web resource**	**Description**	**Availability**	**References**
ModBase	A database of annotated comparative protein structure models and associated resources	https://modbase.compbio.ucsf.edu/modbase-cgi/index.cgi	Pieper et al., 2011
SwissModel Repository	A database of annotated 3D protein structure models generated by the SWISS-MODEL homology-modeling pipeline	https://swissmodel.expasy.org/repository	Bienert et al., 2017

Once modeled, each of the protein structure models undergoes a rigorous quality assessment by employing methods such as NDOPE, GA341 (Shen and Sali, 2006), GOAP (Zhou and Skolnick, 2011), SOAP (Webb and Sali, 2016), Molprobity (Chen et al., 2010) and secondary structure agreement score (Eramian et al., 2006). Models with extensive chain clashes, poorly resolved loops and improperly fitted ligands are ranked low in the consensus quality scoring process described in CHOPIN—a web resource for structural and functional proteome of *Mycobacterium tuberculosis* (Ochoa-Montaño et al., 2015).

**FIGURE 1:**
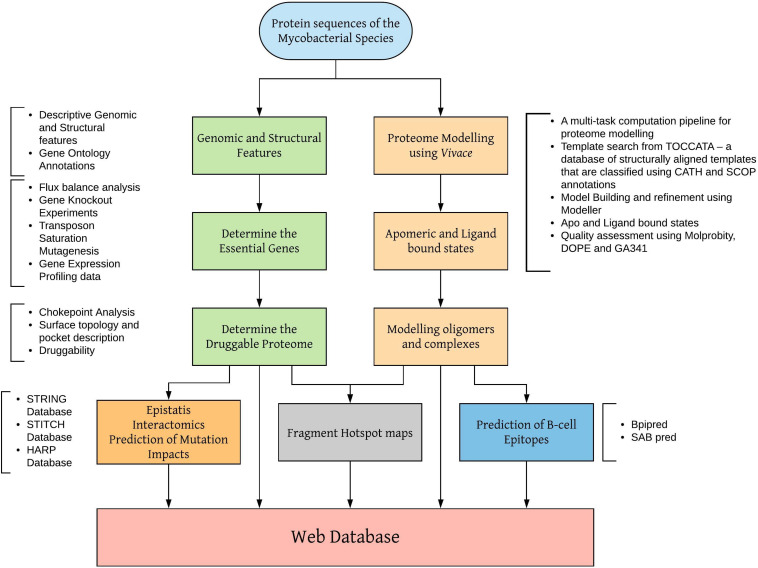
Workflow for modeling mycobacterial proteomes and developing web databases.

*Vivace* is being used to model the proteome of *M. leprae.* Sequence and structural features at the genome-scale are being analyzed to identify essential enzymes that drive chokepoint metabolic reactions. Models in apomeric, ligand-bound and oligomeric (discussed in the later sections) states are being generated and analyzed for surface topology, cavities (Binkowski et al., 2003) and fragment hotspots (sites for potential small molecule binding) (Radoux et al., 2016). The schematic workflow shown in Figure 1 illustrates the modeling procedures adopted by our group to model proteomes of mycobacterial pathogens.

### Approaches to Predict Homo/Hetero-Oligomeric Complexes

Protein-protein interactions (homo/hetero) govern a majority of the cellular processes. Structure determination of these complexes is crucial for understanding their functions. Usually, the experimental techniques used to unravel interacting protein partners are time consuming, challenging and expensive. There have been significant advances in the development of computational methods and tools to identify interacting pairs and predict the structures of protein-protein complexes (Das and Chakrabarti, 2021).

The computational tools for predicting protein-protein interactions developed over the years can be classified into the knowledge-based or *de novo* prediction methods. If the structures of the interacting partners are known, the interactions can be predicted using template-based, or template free and/or restraint-based docking. Template-based docking can provide the multi-component modeled complex but requires the presence of multi-component template structures (Ogmen et al., 2005; Mukherjee and Zhang, 2011). If the homologous multi-component template is unavailable, protein-protein docking approaches can be used to sample the conformational space and predict the docked complexes which are further scored using different schemes to discriminate native-like conformations from a pool of docked solutions. These different approaches for computational modeling of protein interactions were recently reviewed by Soni and Madhusudhan (2017).

Recently, tools have been developed which can make use of the wealth of sequence information available for protein sequences to predict/model interactions accurately. Machine learning approaches including deep learning have played a significant role in training models which can predict the interactions using the features derived from protein sequences alone (Huang Y.-A. et al., 2016; Du et al., 2017; Sun et al., 2017; Chen et al., 2019). The inspection of co-evolving sites in two protein partners can provide strong signals to elucidate interacting partners (Yu et al., 2016). A recent method CoFex (Hu and Chan, 2017) used co-evolutionary features to predict protein interactions. Ensemble based approaches which use multiple machine learning methods to vote for predictions have been reported to achieve high sensitivity and accuracy (Zhang et al., 2019; Li et al., 2020). Deep learning has also been employed to train a convolutional neural network (CNN) to predict the protein interacting pairs with high accuracy (Wang et al., 2019; Torrisi et al., 2020).

However, *in-silico* approaches can often give false positive or negative results as well, hence one also needs validation strategies to assess the quality of predicted interactions. Efforts in the community such as CASP (Critical Assessment of Structure Prediction) and CAPRI (Critical Assessment of Prediction of Interfaces) competitions, aim to assess the field and the state-of-the-art methods and their ability to “correctly” model protein structures and their interactions, respectively. They define and use multiple scores for assessing the quality of protein structure and interfaces in the modeled complexes. CASP14 is the present ongoing competition, where deep learning approach-AlphaFold2 has outperformed and were able to accurately predict the protein structures (AlQuraishi, 2019).

**FIGURE 2:**
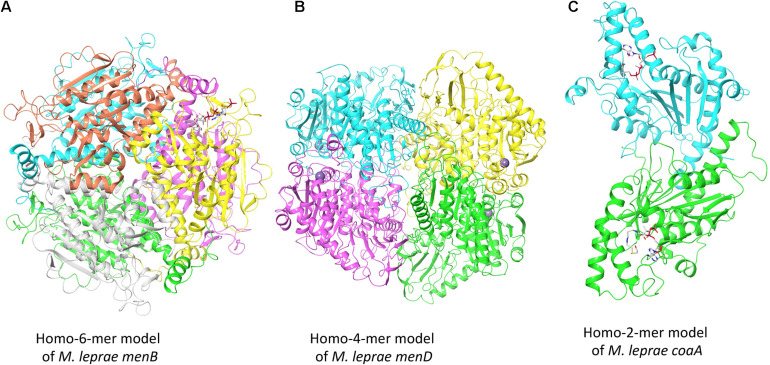
Oligomeric models of three potential drug targets in *M. leprae.* **(A)** The homohexameric model of *M. leprae menB* complexed with Salicylyl CoA. **(B)** The homotetrameric model of *M. leprae menD* bound to magnesium ions. **(C)** The homodimeric model of *M. leprae coaA* in complex with coenzyme A derivative.

To illustrate the modeling process adopted by *Vivace*, Figure 2 depicts the models of three potential drug targets in *M. leprae*, the *menB* [1,4-dihydroxy-2-naphthoyl-CoA synthase (ML2263)], *menD* [2-succinyl-5-enolpyruvyl-6-hydroxy-3-cyclohexene-1-carboxylate synthase (ML2270)] and *coaA* [Pantothenate kinase (ML1954)].

The gene product of *menB* converts o-succinylbenzoyl-CoA (OSB-CoA) to 1,4-dihydroxy-2-naphthoyl-CoA (DHNA-CoA) and its homologue in *M. tuberculosis* (Rv0548c) is reported as essential (DeJesus et al., 2017). We built the model using the structure of its orthologous protein in *M. tuberculosis* (PDB Id: 4QII) as the template with sequence identity of 93% and sequence coverage of 100% (Figure 2A). The gene product of *menD* catalyzes the thiamine diphosphate-dependent decarboxylation of 2-oxoglutarate. Its homologue in *M. tuberculosis* (Rv0555) is noted to be essential for bacterial survival. We modeled *menD* of *M. leprae* using the structure of the *M. tuberculosis* orthlogue (PDB Id: 5ESD) as the template with the sequence identity of 86% and sequence coverage of 99% (Figure 2B). Finally, we modeled *coaA* which synthesizes coA from (R)—Pantothenate. CoaA has been recognized as a drug target in tuberculosis (Chiarelli et al., 2018). We modeled *coaA* using its orthologue in *M. tuberculosis* (PDB Id: 2GET) as the template with sequence identity of 93% and sequence coverage of 98% (Figure 2C).

### Structural Implications of Substitution Mutations

Development of drug resistance is recognized as one of the major hurdles to disease management and control. For *M. leprae*, it is even more challenging as it relies on mouse footpad models (Vedithi et al., 2018). Antimicrobial resistance was noted in Dapsone, Rifampicin and Ofloxacin (a second-line drug). Treating and managing the disease is a big hurdle due to emerging drug resistance for these three drugs and lack of alternative effective treatments.

The drug-resistance mutations when mapped on to the three-dimensional structure of the drug target can provide crucial insights into effects on protein structure and function. There are several available software/web servers that can predict the impacts of mutation on protein stability and interactions with other proteins, ligands and nucleic acids. We have provided a list of some of the commonly used software tools for investigating the effects of mutations on protein structure and function (Table 4).

**TABLE 4:** Some of the commonly used tools for predicting the effect of mutations on protein structure and function.

**Software**	**Description**	**Availability**	**References**
SIFT	Amino acid substitution effect on protein function	https://sift.bii.a-star.edu.sg/	Ng and Henikoff, 2003
PolyPhen-2	Amino acid substitution effect on protein structure and function using protein sequence	http://genetics.bwh.harvard.edu/pph2/	Adzhubei et al., 2010
SNPs3D	Amino acid substitution effect on protein structure and function using SVM based model	http://snps3d.org/	Yue et al., 2006
MutPred2	Machine learning approach to quantify pathogenicity of mutation	http://mutpred.mutdb.org/index.html	Pejaver et al., 2020
PROVEAN	Impact of mutation on protein function by using multiple sequence alignment	http://provean.jcvi.org/index.php	Choi and Chan, 2015
mCSM	Effect of mutation on protein structure and interactions using graph-based signatures	http://biosig.unimelb.edu.au/mcsm/	Pires et al., 2014a
SDM2	Effect of mutation on protein structure and interactions using environment-specific amino-acid substitution frequencies	http://marid.bioc.cam.ac.uk/sdm2	Pandurangan et al., 2017, 2
DUET	Consensus prediction of mCSM and SDM2 for protein stability	http://biosig.unimelb.edu.au/duet/	Pires et al., 2014b
PoPMuSiC-2	Effects of mutation on protein stability using statistical potentials	http://dezyme.com/en/Services	Dehouck et al., 2009
FoldX	Change in free energy using force fields-based method	http://foldxsuite.crg.eu/	Schymkowitz et al., 2005
Hunter	Predicting protein stability upon mutation using side chain interactions	http://bioinfo41.weizmann.ac.il/hunter/	Potapov et al., 2010
MAESTRO	Measures changes in free energy upon mutation using machine learning	https://pbwww.che.sbg.ac.at/?page_id=416	Laimer et al., 2015
I-Mutant3.0	SVM based prediction of protein stability change upon mutation using either sequence and/or structure	http://gpcr2.biocomp.unibo.it/cgi/predictors/I-Mutant3.0/I-Mutant3.0.cgi	Capriotti et al., 2008
MUPro	SVM and neural network-based prediction of changes in protein stability	http://mupro.proteomics.ics.uci.edu/	Cheng et al., 2006
iStable	Change in free energy using SVM based predictor	http://predictor.nchu.edu.tw/istable/	Chen et al., 2013
MutaBind	Change in free energy using force fields, statistical potentials and side-chain optimisation methods	https://www.ncbi.nlm.nih.gov/research/mutabind/index.fcgi/	Li et al., 2016
BeAtMuSiC	Impact of mutations on protein-protein interactions using statistical potentials	http://babylone.ulb.ac.be/beatmusic/	Dehouck et al., 2013
SNAP2	Predict functional impacts of mutations using neural network-based model	https://rostlab.org/services/snap2web/	Hecht et al., 2015
Envision	Supervised, stochastic gradient boosting algorithm to quantify the effect of mutation	https://envision.gs.washington.edu/shiny/envision_new/	Gray et al., 2018
EVmutation	Unsupervised statistical method to predict effect of mutations using residue dependencies between positions	https://marks.hms.harvard.edu/evmutation/	

Our own group have developed over the past decade the mCSM suite of computer programmes that use ML/AI approaches to predict the impacts of amino acid mutations not only on protomer stability (Pires et al., 2014a) but also on protein-protein, protein nucleic acid and protein-ligand interactions (Pires et al., 2016; Pires and Ascher, 2017). Recently, there have been further developments in the field where machine learning (ML)-based methods are gaining popularity. Many more recent ML methods also use features derived from protein structure and/or sequence to predict the effect of mutations (Hopf et al., 2017). A recent review, summarizes the performance of different ML methods and emphasizes the need for selecting reliable training dataset and informative features (Fang, 2020). Deep learning is an advanced training which is composed of multiple layers to learn different features from the input data and is proven to learn from the high-dimensional data. Recently, a method called DeepCLIP (Grønning et al., 2020) has been proposed which can predict protein binding to RNA using only sequence data. Another recently developed deep learning framework-MuPIPR (Zhou et al., 2020) (Mutation Effects in Protein–protein Interaction Prediction Using Contextualized Representations), can predict the effects of mutation on protein-protein interactions in terms of changes in buried surface area and binding affinity.

### *In-silico* Saturation Mutagenesis

Using the tools described above, computational efforts exploiting recent growth in the availability of computing power can be immensely helpful to perform saturation mutagenesis, which can be used as a surveillance tool for drug resistance in leprosy. These mutational scanning exercises can provide crucial insights into the structure-function relationships by exploring all possible substitutions at a given site. This can provide a glimpse into the functional consequences of mutations in antimicrobial-resistance phenotypes. The extensive quantitative data from computational saturation mutagenesis experiments can guide experimental approaches and prove helpful for validation and/or engineering purposes. Recently published HARP (a database of Hansen’s Disease Antimicrobial Resistance Profiles) database (Vedithi et al., 2020) is a comprehensive repository of *in-silico* mutagenesis experiments for three important drug targets for *M. leprae* namely dihydropteroate synthase, RNA polymerase and DNA gyrase. A consensus impact for all the possible mutations on protein stability and function of these drug targets is provided in this database.

### Druggability

**FIGURE 3:**
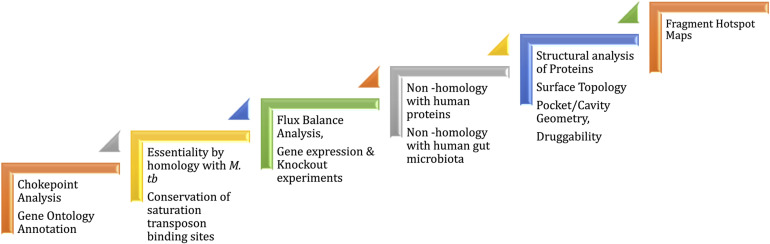
Workflow for drug target prioritization in *M. leprae.*

*Mycobacterium leprae* genome is reduced to 3,268,203 bp preserving only 1,614 ORFs (Cole et al., 2001; Liu et al., 2004) of the Mycobacterial genus. The genome reduction is due to evolutionary adaptation of this intracellular obligate bacillus to Schwann and macrophages cells. Gene essentiality in *M. leprae* is deciphered based on essentiality of homologous genes, mainly in *M. tuberculosis* that are determined by experiments (Sassetti et al., 2003; DeJesus et al., 2017). Because of the evolutionary loss of non-essential genes by pseudogenization, only 65% of the existing total of *M. leprae* genes have been demonstrated to be essentials (Borah et al., 2020). In order to identify potential drug targets, a chokepoint reaction analysis helps to find proteins that are either consumers of unique substrates, or are unique producers of metabolites. It is predicted that the inhibition of chokepoint proteins produces an interruption of essential cell functions (Yeh et al., 2004). Determining the druggability of protein targets is important to avoid intractable targets. A druggable protein has the ability to bind with high affinity to a drug. In leprosy, the dihydropteroate synthase (DHPS), RNA polymerase (RNAP) and DNA gyrase (GYR) are known druggable proteins as they are the targets of Dapsone, Rifampicin and Ofloxacin, respectively. Nevertheless, protein druggability properties can be predicted by different bioinformatics tools based on the 3D structure/model of the protein. For example, the α-1,2-mannosyltransferase and mannosyltransferase proteins related to lipoarabinomannan pathway were identified as a possible drug targets using CASTp (Computer Altas of Surface Topography of proteins) (Gupta et al., 2020). CASTp determines the volume and the area of each cavity and pocket. Furthermore, for each pocket the solvent accessible surface and the molecular surface are calculated. Small-molecule virtual screening is another *in-silico* strategy used to ascertain druggability of the protein target. This approach provides an understanding of the physicochemical features of the binding sites and potential ligands that bind at these sites. In *Mycobacterium tuberculosis*, 2,809 proteins are identified as druggable using this *in-silico* approach (Anand and Chandra, 2014). Mammalian cell entry proteins of the class *mce1A-E* have been reported in *M. leprae* to facilitate bacterial entry into human nasal epithelial cells (Fadlitha et al., 2019). *Mce1A* has a significant role in the cell predilection and a comprehensive understanding of the structure and druggability of this protein can provide insights into host pathogen interactions and transmission in leprosy (Sato et al., 2007). In the case of ML2177c, this gene encodes for uridine phosphorylase and shows significantly high expression during leprosy infection. This is predicted as druggable using fragment-hotspot-map analysis (Malhotra et al., 2017). The fragment hotspots contain a juxtaposition of charge and lipophilicity that are essential for effective ligand binding through both enthalpic and entropic contributions. The hotspot map software uses different molecular probes (toluene, aniline and phenol) to calculate affinity maps that provide a visual guide of the pocket (Radoux et al., 2016). Figure 3 illustrates the recommended pathway to target prioritization in mycobacterial drug discovery.

### Structure-Guided Virtual Screening

Structure-guided virtual screening is a cost-effective computational tool for preliminary screening of proteins that are potential drug targets with chemical libraries ranging from small core fragments to large macrocyclic compounds in size and scaling from a few hundred molecules to billions (used in ultra-large-scale virtual screening campaigns). Since physical synthesis of drug molecules is not required, millions of virtual chemical entities can be swiftly docked into the active site of the protein structure/model and appropriate chemical scaffolds that fit with high scores and form relevant stabilizing interactions can be shortlisted for experimental validations. Virtual screening can be applied to novel drug discovery and also in drug repositioning experiments (screening with existing approved drugs to identify new target-protein interactions). A repurposing screen of LipU, a lipolytic protein that is conserved across mycobacterial species and noted to be essential for survival of *M. leprae*, revealed high docking scores for anti-viral drugs and anti-hypertensive (Kaur et al., 2019). Molecular docking software, such as Glide (Friesner et al., 2006), CCDC-GOLD (Jones et al., 1997), Autodock (Goodsell and Olson, 1990), Ledock (Wang et al., 2016), FlexX (Kramer et al., 1999), and SwissDock (Grosdidier et al., 2011) are used in virtual screening campaigns. Each algorithm has a unique scoring function to assess the fitness, number of stable interatomic interactions, and changes in energy landscape.

## Discussion and Conclusion

Here, we have reviewed the tools and the advances made in protein structure prediction, modeling of genomes and impacts of amino acid replacements on protein structure and function. We have discussed these areas particularly focusing on the mycobacterial genomes, more specifically *M. leprae*. Given the paucity of information related to structural proteomic studies in leprosy, we discussed a multi-task protein modeling pipeline that enables proteome-scale template-based modeling of individual proteins encoded by various annotated genes in *M. leprae*. Homology-based structural and functional annotation of these protein models (Ochoa-Montaño et al., 2015; Skwark et al., 2019) using appropriate computational tools for modeling and druggability assessment can expedite characterization of the structural proteome of *M. leprae* and accelerate structure-guided novel drug discovery to combat nerve damage associated with leprosy.

Using the latest advancements in the field of protein structure bioinformatics, we describe our attempts to perform proteome scale modeling of mycobacterial genomes using in-house databases and pipelines. The modeled protein monomers or (homo/hetero) oligomers are subjected to multiple state-of-the-art validation scores. These models can be very helpful and provide useful insights to understand protein structure and function, identify drug targets and unravel their functional roles in the pathogen. The structures of selected drug targets can also help in experimental design and prioritizing the protein targets for validation.

The emergence of drug resistance to the multidrug therapy is a major challenge in treating mycobacterial infections especially leprosy where structural features of drug-target interactions are poorly understood. To complement the computational findings, our group has employed cryoEM methods to understand the impact of mutations on the structure of catalase peroxidase in *M. tuberculosis* (Munir et al., 2019, 2021). Protein sequences and structures can be used to model the impacts of drug resistance mutation on protein structure and function. We have described various software available for predicting the impacts of mutations using protein sequence or structure or both. *In-silico* saturation mutagenesis experiments can guide the experimental design and help in saving the time and labor required to conduct laboratory experiments on animal models. Structure-based drug design (Blundell et al., 2002; Blundell and Patel, 2004) is a way forward and is a promising approach to design new drugs and treatments.

## Author Contributions

SV, SM, and MADGE conducted the review and written the manuscript. MM, PT, and TB reviewed the manuscript and provided necessary additions to the text. All authors contributed to the article and approved the submitted version.

## Conflict of Interest

The authors declare that the research was conducted in the absence of any commercial or financial relationships that could be construed as a potential conflict of interest.
